# Retrospective Camera‐Based Respiratory Gating in Clinical Whole‐Heart 4D Flow MRI

**DOI:** 10.1002/jmri.27564

**Published:** 2021-03-10

**Authors:** Lukas M. Gottwald, Carmen P.S. Blanken, João Tourais, Jouke Smink, R. Nils Planken, S. Matthijs Boekholdt, Lilian J. Meijboom, Bram F. Coolen, Gustav J. Strijkers, Aart J. Nederveen, Pim van Ooij

**Affiliations:** ^1^ Radiology and Nuclear Medicine Amsterdam Amsterdam University Medical Centers, location AMC The Netherlands; ^2^ MR R&D—Clinical Science Philips Healthcare Best The Netherlands; ^3^ Biomedical Engineering Eindhoven University of Technology Eindhoven The Netherlands; ^4^ Magnetic Resonance Systems Lab, Department of Imaging Physics Delft University of Technology Delft The Netherlands; ^5^ Cardiology Amsterdam University Medical Centers Amsterdam The Netherlands; ^6^ Biomedical Engineering and Physics Amsterdam University Medical Centers Amsterdam The Netherlands

**Keywords:** Camera, navigator, respiratory gating, 4D flow MRI

## Abstract

**Background:**

Respiratory gating is generally recommended in 4D flow MRI of the heart to avoid blurring and motion artifacts. Recently, a novel automated contact‐less camera‐based respiratory motion sensor has been introduced.

**Purpose:**

To compare camera‐based respiratory gating (CAM) with liver‐lung‐navigator‐based gating (NAV) and no gating (NO) for whole‐heart 4D flow MRI.

**Study Type:**

Retrospective.

**Subjects:**

Thirty two patients with a spectrum of cardiovascular diseases.

**Field Strength/Sequence:**

A 3T, 3D‐cine spoiled‐gradient‐echo‐T1‐weighted‐sequence with flow‐encoding in three spatial directions.

**Assessment:**

Respiratory phases were derived and compared against each other by cross‐correlation. Three radiologists/cardiologist scored images reconstructed with camera‐based, navigator‐based, and no respiratory gating with a 4‐point Likert scale (qualitative analysis). Quantitative image quality analysis, in form of signal‐to‐noise ratio (SNR) and liver‐lung‐edge (LLE) for sharpness and quantitative flow analysis of the valves were performed semi‐automatically.

**Statistical Tests:**

One‐way repeated measured analysis of variance (ANOVA) with Wilks's lambda testing and follow‐up pairwise comparisons. Significance level of *P* ≤ 0.05. Krippendorff's‐alpha‐test for inter‐rater reliability.

**Results:**

The respiratory signal analysis revealed that CAM and NAV phases were highly correlated (*C* = 0.93 ± 0.09, *P* < 0.01). Image scoring showed poor inter‐rater reliability and no significant differences were observed (*P* ≥ 0.16). The image quality comparison showed that NAV and CAM were superior to NO with higher SNR (*P* = 0.02) and smaller LLE (*P* < 0.01). The quantitative flow analysis showed significant differences between the three respiratory‐gated reconstructions in the tricuspid and pulmonary valves (*P* ≤ 0.05), but not in the mitral and aortic valves (*P* > 0.05). Pairwise comparisons showed that reconstructions without respiratory gating were different in flow measurements to either CAM or NAV or both, but no differences were found between CAM and NAV reconstructions.

**Data Conclusion:**

Camera‐based respiratory gating performed as well as conventional liver‐lung‐navigator‐based respiratory gating. Quantitative image quality analysis showed that both techniques were equivalent and superior to no‐gating‐reconstructions. Quantitative flow analysis revealed local flow differences (tricuspid/pulmonary valves) in images of no‐gating‐reconstructions, but no differences were found between images reconstructed with camera‐based and navigator‐based respiratory gating.

**Level of Evidence:**

3

**Technical Efficacy:**

Stage 2

Whole‐heart 4D flow MRI is an emerging technique with important application in the diagnosis and risk assessment of structural heart diseases via quantification of hemodynamic parameters and intracardiac flow visualization.^1^, ^2^, ^3^, ^4^, ^5^, ^6^ To avoid blurring and motion artifacts, respiratory gating is generally recommended in 4D flow MRI.^1^, ^2^, ^7^

Several methods have been developed to track patient breathing during image acquisition. The 4D flow consensus statement paper recommends the use of a belt or a navigator.^1^, ^8^ The latter involves additional radiofrequency pulses to dynamically track the anatomic motion of usually the liver‐lung boundary.^1^, ^8^ Another option is self‐gating,^9^, ^10^, ^11^, ^12^ in which the respiratory motion information is calculated from the MRI acquisition itself if the k‐space sampling was performed in a certain order and a frequency high enough to capture the respiratory motion. However, this is not the case for standard cartesian 4D flow sequences which are usually used in clinical practice, and, therefore, self‐gating cannot be applied there. In some cases, respiratory gating can be omitted with acceptable quantitative results and image quality.^13^, ^14^, ^15^ However, higher‐resolution 4D flow MRI requires accurate and reliable respiratory gating.^7^

Respiratory motion information can be used prospectively or retrospectively to acquire or accept data only during a time window of minimal respiration‐induced motion, usually at end‐expiration. Prospective gating has the drawback that the scan time is not exactly known a priori and may increase significantly in case of low respiratory gating efficiency.^16^ Retrospective gating requires sufficient oversampling of the data to ensure that enough k‐space points are acquired for reconstruction. The need for oversampling can be reduced by employing efficient k‐space acquisition strategies, including radial or spiral readouts and appropriate reconstruction techniques such as compressed sensing.^10^, ^17^, ^18^

Recently, a novel automated contact‐less camera‐based respiratory motion sensor has been introduced.^19^, ^20^ The input video signal is divided into equal‐sized rectangular blocks, then the blocks containing periodic respiratory motion are identified, weighed, and used to track respiratory motion. This gating technique is easy to use as it requires no additional manual steps such as belt placement or sequence planning and can be used for prospective or retrospective triggering. Harder et al. have demonstrated improved image quality in abdominal MRI with prospective camera‐based respiratory gating compared to belt‐based respiratory gating,^21^ which evoked the questions of how this technique performs in 4D flow MRI.

This study aimed to evaluate camera‐based retrospective respiratory gating for whole‐heart 4D flow MRI in patients with cardiovascular diseases.

## Methods

### Study Cohort

The study cohort consisted of 32 patients (34 ± 18 years, range 9–73 years; 17 male/15 female). Included were all patients that underwent a whole‐heart 4D flow MRI exam between September 2019 and March 2020. This group of patients consisted of a spectrum of cardiovascular diseases, including valvular heart disease, aortic disease, and complex structural heart disease (see Table S1 in the Supplemental Material). The study design was retrospective and data analysis was anonymous, so the requirement for written informed consent was waived by the local ethics committee. Exclusion criteria for quantitative flow analysis were: the field of view did not contain the entire heart, the standard clinical 2D cine images were missing or were of insufficient quality to contour the valves.

### Data Acquisition

All MRI data sets were acquired with a dStream Torso coil on a 3T MR system (Philips Ingenia ElitionX; Philips Medical Systems). In the standard clinical routine protocol of mainly 2D cine MRI scans, pseudo‐spiral compressed sensing accelerated 4D flow MRI scan was performed for each patient.^18^, ^22^ All MRI scans were synchronized with the heartbeat by electrocardiogram‐gating. 4D flow MRI scans were acquired with a gradient‐echo sequence undersampled by a factor of 7.1. Scan parameters were echo time / repetition time / flip angle of 2.0 ms / 4.0 ms / 8°, acquisition and reconstruction voxel size of 2.5 mm isotropic, and velocity encoding in the range of 150–250 cm/s. Acceleration factor, scan time as well as temporal resolution was calculated as a mean over the study cohort.

**FIGURE 1:**
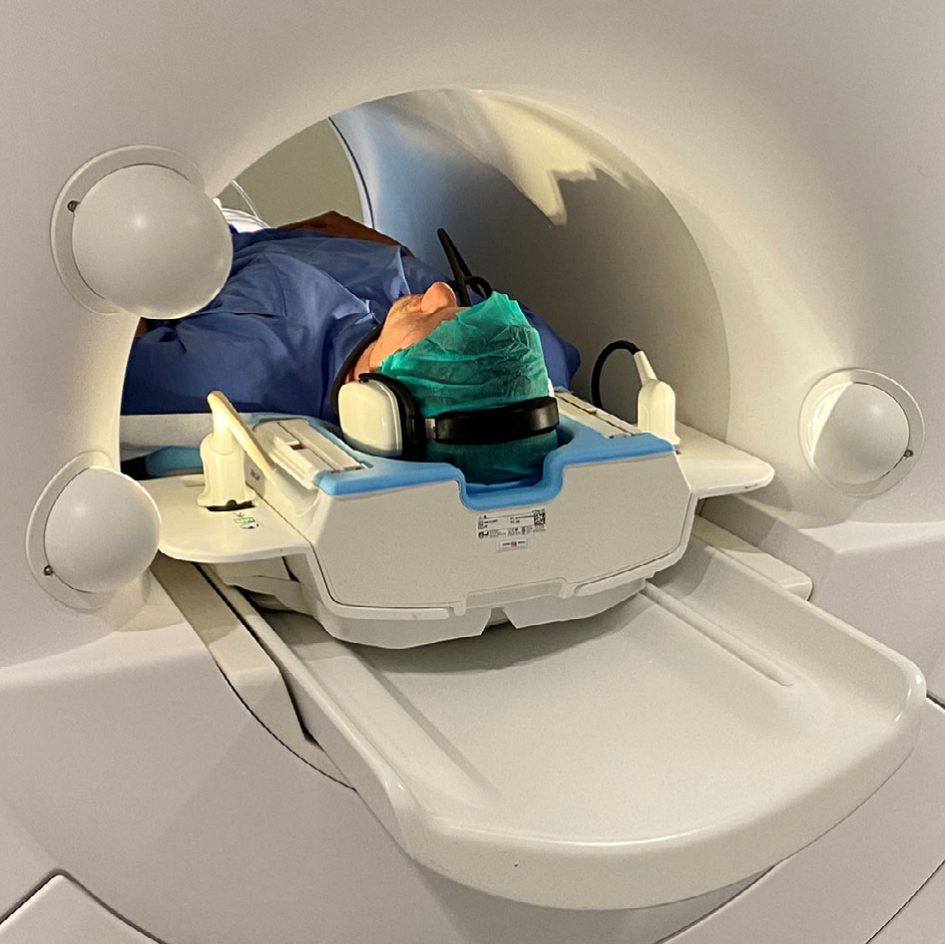
Visualization of the built‐in‐the‐bore vital sign camera (VitalEye, Philips Medical Systems). The camera is inside the top left plastic casing and focuses on the subject's upper body. The other two plastic casings on table height are spotlights. Usage of a head coil instead of a body coil was for demonstration purposes only.

Respiratory motion was measured simultaneously by the conventional liver‐lung‐navigator and a camera sensor (VitalEye, Philips Medical Systems) as shown in Fig. 1. The navigator was placed on the liver‐lung border. The MRI data acquisition was modified for this 4D flow protocol to acquire pencil beam navigators with a sampling frequency of 2 Hz regardless of the cardiac cycle. A built‐in‐the bore camera (uEye, IDS Imaging Development Systems) targeted the upper body, and a fully automated algorithm derived the respiratory signal in real‐time by identifying image blocks that contained the respiratory motion. The camera‐based respiratory signal was streamed to the scanner with a sampling frequency of 20 Hz.^19^

### Respiratory Binning and Data Reconstruction

4D flow data were reconstructed offline using ReconFrame (Gyrotools) in MATLAB (MathWorks) together with the Berkeley Advanced Reconstruction Toolbox^23^ for compressed sensing reconstruction with a sparsifying total variation transform in time.^18^, ^22^ Apart from retrospective cardiac gating, camera‐based respiratory gating (CAM) and navigator‐based respiratory gating (NAV) of the raw data was performed with respiratory phase binning in inspiration and expiration. The expiration phase acceptance was defined at 60%.^24^ Additionally, all 4D flow data sets were also reconstructed with no respiratory gating (NO) representing 100% respiratory phase acceptance. The cardiac cycle was binned into 30 frames. After the reconstruction, phase unwrapping (velocity aliasing correction) of the 4D flow data was automatically performed with a 4D single‐step Laplacian algorithm.^25^

Applied phase binning algorithm: Read in the raw signal, rescale the signal to zero median, smooth signal over 1 second, define the minimal distance between same sign peaks to 45 breath per minute (highest expected breathing frequency), calculate extrema (minima/maxima) with minimal distance: islocalmax (signal, minimal distance) MATLAB function, calculate minimal peak prominence as one fourth of the median maxima–minima distance, calculate extrema (minima/maxima) with minimal distance and minimal peak prominence: islocalmax (signal, minimal distance, and minimal peak prominence) MATLAB function, correct for double extrema in case one minimum is followed by two maximums and vice versa, phase bin the respiratory signal in 100 bins, reject inspiration data (1–40) and accept expiration data (41–100), label the data according to the signal.

### Respiratory Signal Analysis

After the respiratory binning in the reconstruction, the respiratory signals, as well as their corresponding respiratory phases, were extracted from both CAM and NAV. The cross‐correlation of the respiratory phases per subject was calculated to evaluate their similarity. Furthermore, the time shift between the two phases (phase delay) was measured.

### Qualitative Image Analysis

Qualitative image analysis was performed independently and blinded by a radiologist with 15 (RNP), a cardiothoracic radiologist with 8 years (LJM), and a cardiologist with 10 (SMB) years of experience in cardiovascular imaging. Images were provided as transversal magnitude and phase‐contrast cine images at two locations. One location was intersecting the heart chambers and the other was intersecting the great vessels. Image scoring was based on a 4‐point Likert scale: 1 = unusable, 2 = fair, 3 = good, 4 = excellent. Rated were four categories: anatomical structure, flow signal, breathing artifacts, and flow artifacts.

### Quantitative Image Analysis

Quantitative image analysis was performed using the phase‐contrast magnitude images by calculating the signal‐to‐noise ratio (SNR) and the liver‐lung‐edge (LLE) from a 10 × 10 × 30 voxel region‐of‐interest (ROI). The ROI was manually drawn per patient at the liver‐lung border at the expected location of the navigator. In this ROI two transversal slices were selected: one in the liver and another in the lung. The slice in the liver was defined as the signal area and the slice in the lung was defined as the noise area. SNR was defined as the time‐averaged mean signal intensity divided by the time‐averaged SD of the noise. Between the liver and the lung slice in the ROI, 100 line profiles in z‐direction were extracted and fitted on a sigmoid function. LLE was defined by the mean width $\bar{d}$ of all sigmoid functions [voxel].

### Quantitative Flow Analysis

The reconstructed velocity images were processed in Cardiovascular Angiographic Analysis Systems (CAAS; MR Solutions 5.1—4D flow, Pie Medical Imaging) to analyze the transvalvular blood flow of the tricuspid valve (TV), pulmonary valve (PV), mitral valve (MV), and aortic valve (AV). The 2D cine images were used to mark all cardiac valves and track their motion.^26^ The 2D cine and 4D flow MRI images were aligned, and contours were drawn to measure the blood flow across all four heart valves. As parameters of interest, forward flow volume [ml], backward flow volume [ml], regurgitation fraction [ ],^27^ and velocity rate [cm/s] (mean of the contour per time point) per valve were chosen. Moreover, backward flow volumes and regurgitation fraction were compared of a mixed subgroup *n*_2_ containing only valves diagnosed with insufficiency.

### Statistical Analysis

For each sub‐analysis, a one‐way repeated measured analysis of variance (ANOVA) was conducted to evaluate the null hypothesis that there is no change between the three different respiratory gating techniques (CAM, NAV, and NO). Level of significance was defined for *P* < 0.05. Pairwise comparisons were Bonferroni corrected. The Krippendorff's alpha test for ordinal data was used to estimate the inter‐rater reliability alpha (*α*) for the Likert scoring in the qualitative image analysis.^28^ Values were reported as mean ± SD. Additionally, pairwise comparisons for the quantitative image and flow analyses were presented in the form of Bland–Altman plots.

## Results

### Study Cohort and Data Acquisition

The average scan time was 586 ± 103 s, ranging from 397 to 757 s, depending on the field of view needed to cover the patient's heart. Retrospective cardiac binning into 30 frames resulted in a temporal resolution of 28.0 ± 4.7 ms, corresponding to acceleration factors of 10.73 ± 1.08 for CAM, 10.74 ± 1.07 for NAV, and 6.89 ± 0.81 for NO. Nine data sets were excluded from the quantitative flow analysis due to exclusion criteria. The remaining subset *n*_1_ consisted of 23 patients (30 ± 16 years, range 9–73 years; 12 male/11 female). A detailed overview of the patient cohort is provided in Table S1 in the Supplemental Material.

### Respiratory Signal Analysis

**FIGURE 2:**
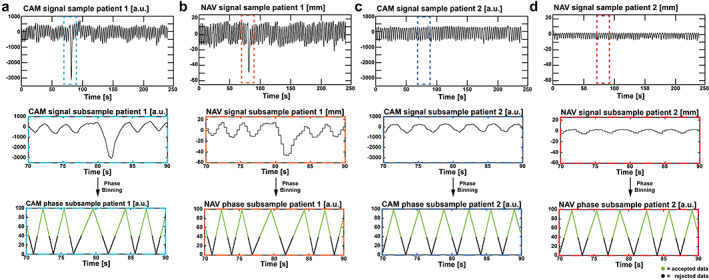
Respiratory signal samples (top) of two patients for both CAM (a, c) and NAV (b, d) are illustrated. Zoomed regions of the respiratory signals are shown in the middle with the corresponding respiratory phases after phase binning on the bottom. Green respiratory phases indicate accepted data points.

The respiratory signal analysis revealed that the camera‐ and navigator‐derived respiratory phases were highly correlated as their cross‐correlation was *C*_phase_ = 0.93 ± 0.09 (*P* < 0.01). The significance was tested for the hypothesis that the cross‐correlation is <0.5 (no strong correlation). The corresponding phase delays between the camera and navigator phase were *d*_phase_ = 0 ± 63 ms. In Fig. 2, respiratory signal samples of two patients are shown for CAM and NAV. Both patients' CAM signals have similar ranges, but their NAV signal amplitude ranges differ approximately by a factor of 3. Zoomed regions of the respiratory signals are shown in the middle plots, and the corresponding respiratory phases show high correlation after binning on the bottom plots. Figure S1 in the Supplemental Material contains both the CAM and NAV signals for all patients as well as signal boxplots over the entire cohort. Calculated in the boxplots were the inter‐quartile ranges, upper and lower whiskers (W_up_, W_low_) as well as minima and maxima. While the ratio of (W_up_ − W_low_)_CAM_/(W_up_ − W_low_)_NAV_ was 87, the ratio of (maxima‐minima)_CAM_/(maxima‐minima)_NAV_ was 161, highlighting the signal amplitude differences from the outliers, which are not noticeable after phase binning.

### Qualitative Image Analysis

**FIGURE 3:**
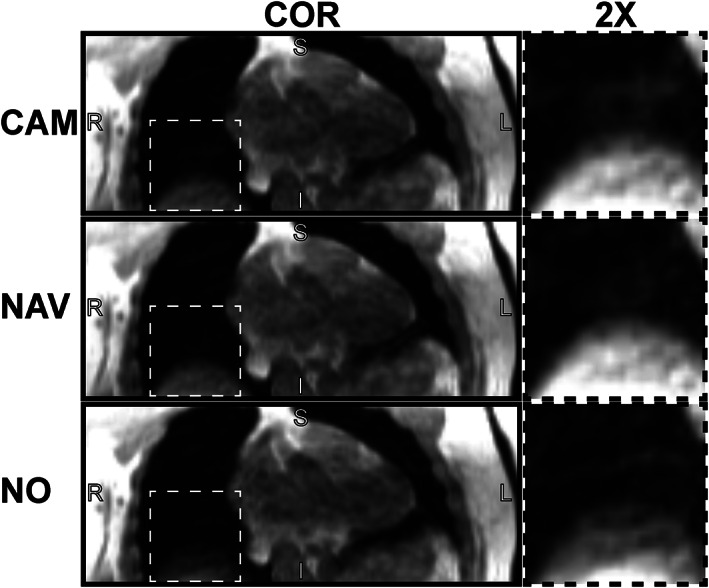
Samples of coronal images with camera‐based (CAM), navigator‐based (NAV), and no respiratory gating (NO). A region (dotted box) around the liver‐lung border is 2‐fold magnified on the right.

Image samples of 4D flow data sets reconstructed with NAV, CAM, and NO are shown in Fig. 3. No significant differences between the three reconstructions could be found for anatomical structure (Wilks' lambda = 0.99, *F*(2,92) = 0.44, *P* = 0.65, *η* ^2^ = 0.01), flow signal (Wilks' lambda = 0.96, *F*(2,92) = 1.86, *P* = 0.16, *η* ^2^ = 0.04), breathing artifacts (Wilks' lambda = 1.00, *F*(2,92) = 0.50, *P* = 0.95, *η* ^2^ = 0.001), and flow artifacts (Wilks' lambda = 0.99, *F*(2,92) = 0.41, *P* = 0.66, *η* ^2^ = 0.01). Inter‐rater reliability was low over all categories, i.e., anatomical structure with *α* = 0.46, flow signal with *α* = 0.24, breathing artifacts with *α* = 0.39, and flow artifacts with *α* = 0.24. All pairwise comparisons are listed in Table S2 and illustrated in Fig. S2 in the Supplemental Material.

### Quantitative Image Analysis

**FIGURE 4:**
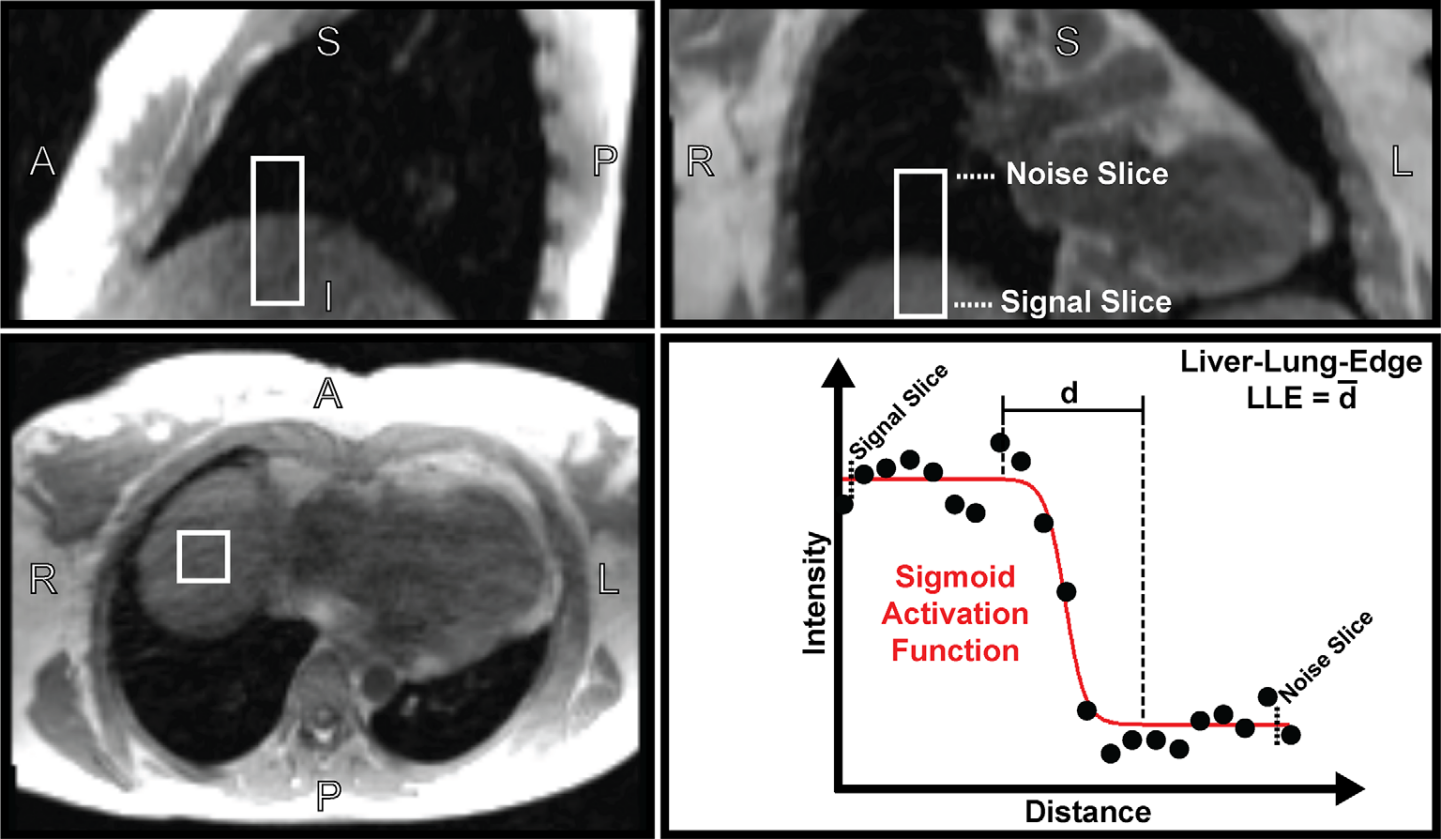
Quantitative image analysis illustration. The ROI (white box) of 10 × 10 × 30 voxels was manually defined per patient at the liver‐lung border at the expected location of the navigator (see sagittal image, top left; and transversal image bottom left). In this ROI two slices were selected; one was entirely in the liver and the other entirely in the lung. The slice in the liver was defined as the signal area and the slice in the lung was defined as the noise area (see coronal image, top right). Between the liver and the lung slice in the ROI, 100 line profiles were extracted and fitted on a sigmoid function. Liver‐lung‐edge (LLE) was defined by the mean width of the sigmoid activation functions.

The quantitative image quality comparison, illustrated in Fig. 4, showed that reconstructions with NAV and CAM were superior to NO in terms of SNR (Wilks' lambda = 0.77, *F*(2,92) = 4.63, *P* = 0.02, *η* ^2^ = 0.24), as well as LLE (Wilks' lambda = 0.40, *F*(2,92) = 22.31, *P* < 0.01, *η* ^2^ = 0.60). Follow up pairwise comparison indicated no significant differences for NAV‐vs‐CAM in SNR (*P* = 1.0) and LLE (*P* = 1.0), whereas the comparison of CAM‐vs‐NO as well as NAV‐vs‐NO showed a significant difference for SNR of 1.69 ± 0.57 (*P* = 0.02) and 1.53 ± 0.51 voxel (*P* = 0.02), and LLE of −1.82 ± 0.29 voxel (*P* < 0.01) and −1.80 ± 0.27 (*P* < 0.01). An example of an increased LLE for NO compared to CAM and NAV is shown in Fig. 3, in which the larger LLE is visible in the blurred liver‐lung border. The SNR and LLE pairwise comparisons are listed in Table 1 and illustrated in Fig. S3 in the Supplemental Material.

**TABLE 1:** Pairwise Comparisons of Quantitative Image Quality Analysis

*X*	*Y*	Mean Difference (*X* − *Y*)	Standard Error	Significance^a^	95% Confidence Interval for Difference^a^	
					Lower Bound	Upper Bound
SNR [ ], *N* = 32						
CAM	NAV	0.15	0.28	1.00	−0.56	0.87
	NO*	1.69	0.57	0.02	0.24	3.13
NAV	CAM	−0.15	0.28	1.00	−0.87	0.56
	NO*	1.53	0.51	0.02	0.25	2.82
NO	CAM*	−1.69	0.57	0.02	−3.13	−0.24
	NAV*	−1.53	0.51	0.02	−2.82	−0.25
LLE [voxel], *N* = 32						
CAM	NAV	−0.02	0.14	1.00	−0.37	0.33
	NO*	−1.82	0.29	<0.01	−2.55	−1.09
NAV	CAM	0.02	0.14	1.00	−0.33	0.37
	NO*	−1.80	0.27	<0.01	−2.48	−1.12
NO	CAM*	1.82	0.29	<0.01	1.09	2.55
	NAV*	1.80	0.27	<0.01	1.12	2.48

^a^ Adjustment for multiple comparisons: Bonferroni.

* The mean difference is significant at the 0.05 level.

### Quantitative Flow Analysis

The quantitative flow analysis for the TV showed no significant difference between the three respiratory gated reconstructions for forward flow volume (Wilks' lambda = 0.82, *F*(2,21) = 2.34, *P* = 0.12, *η* ^2^ = 0.18). However, a significant difference was found for backward flow volume (Wilks' lambda = 0.70, *F*(2,21) = 4.60, *P* = 0.02, *η* ^2^ = 0.30), regurgitation fraction (Wilks' lambda = 0.66, *F*(2,21) = 5.54, *P* = 0.01, *η* ^2^ = 0.35), and velocity rate (Wilks' lambda = 0.95, *F*(2,687) = 17.30, *P* < 0.01, *η* ^2^ = 0.48). Follow up pairwise comparison indicated a significant difference only for CAM‐vs‐NO in backward flow volume −1.44 ± 0.48 ml (*P* = 0.02), CAM‐vs‐NO in regurgitation fraction −0.016 ± 0.005 (*P* = 0.02) and CAM‐vs‐NO in velocity rate 0.45 ± 0.12 cm/s (*P* < 0.01). Thus, NO data sets had higher backward flow volume, large regurgitation fraction, and lower velocity rate compared to CAM data sets. The TV pairwise comparisons are listed in Table 2 and illustrated in Fig. S4 in the Supplemental Material.

**TABLE 2:** Pairwise Comparisons of Tricuspid Valve (TV) Quantitative Flow Analysis

*X*	*Y*	Mean Difference (*X* − *Y*)	Standard Error	Significance^a^	95% Confidence Interval for Difference^a^	
					Lower Bound	Upper Bound
Forward flow volume (TV) [ml], *N* = 23						
CAM	NAV	−0.48	0.63	1.00	−2.11	1.15
	NO	0.93	0.52	0.26	−0.42	2.28
NAV	CAM	0.48	0.63	1.00	−1.15	2.11
	NO	1.41	0.70	0.17	−0.40	3.22
NO	CAM	−0.93	0.52	0.26	−2.28	0.42
	NAV	−1.41	0.70	0.17	−3.22	0.40
Backward flow volume (TV) [ml], *N* = 23						
CAM	NAV	−0.54	0.24	0.10	−1.15	0.07
	NO*	−1.44	0.48	0.02	−2.67	−0.20
NAV	CAM	0.54	0.24	0.10	−0.07	1.15
	NO	−0.90	0.39	0.09	−1.90	0.11
NO	CAM*	1.44	0.48	0.02	0.20	2.67
	NAV	0.90	0.39	0.09	−0.11	1.90
Regurgitation fraction (TV) [ ], *N* = 23						
CAM	NAV	−0.006	0.002	0.07	−0.013	0.000
	NO*	−0.016	0.005	0.02	−0.030	−0.002
NAV	CAM	0.006	0.002	0.07	0.000	0.013
	NO	−0.010	0.005	0.17	−0.023	0.003
NO	CAM*	0.016	0.005	0.02	0.002	0.030
	NAV	0.010	0.005	0.17	−0.003	0.023
Velocity rate (TV) [cm/s], *N* = 690						
CAM	NAV	0.23	0.10	0.09	−0.03	0.48
	NO*	0.45	0.12	0.00	0.16	0.74
NAV	CAM	−0.23	0.10	0.09	−0.48	0.03
	NO	0.22	0.12	0.21	−0.07	0.51
NO	CAM*	−0.45	0.12	<0.01	−0.74	−0.16
	NAV	−0.22	0.12	0.21	−0.51	0.07

* The mean difference is significant at the 0.05 level.

^a^ Adjustment for multiple comparisons: Bonferroni.

For the PV, no significant difference was observed for backward flow volume (Wilks' lambda = 0.90, *F*(2,20) = 1.06, *P* = 0.36, *η* ^2^ = 0.01) and regurgitation fraction (Wilks' lambda = 0.97, *F*(2,20) = 0.32, *P* = 0.73, *η* ^2^ = 0.03). However, a significant difference was observed for forward flow volume (Wilks' lambda = 0.38, *F*(2,20) = 16.69, *P* < 0.01, *η* ^2^ = 0.63) and velocity rate (Wilks' lambda = 0.97, *F*(2,657) = 11.07, *P* < 0.01, *η* ^2^ = 0.03). Follow up pairwise comparison showed a significant difference in forward flow volume for NAV‐vs‐NO of −1.87 ± 0.32 ml (*P* < 0.01) and CAM‐vs‐NO of −1.65 ± 0.57 ml (*P* = 0.03), and velocity rate for CAM‐vs‐NO of −0.54 ± 0.17 cm/s (*P* < 0.01) and NAV‐vs‐NO of −0.58 ± 0.16 cm/s (*P* < 0.01). Thus, NO data sets had lower forward flow volume and lower velocity rate compared to CAM as well as NAV data sets. The PV pairwise comparisons are listed in Table 3 and illustrated in Fig. S5 in the Supplemental Material.

**TABLE 3:** Pairwise Comparisons of Pulmonary Valve (PV) Quantitative Flow Analysis

*X*	*Y*	Mean difference (*X* − *Y*)	Standard Error	Significance^a^	95% Confidence Interval for Difference^a^	
					Lower Bound	Upper Bound
Forward flow volume (PV) [ml], *N* = 22						
CAM	NAV	0.21	0.55	1.00	−1.22	1.64
	NO*	−1.65	0.57	0.03	−3.14	−0.16
NAV	CAM	−0.21	0.55	1.00	−1.64	1.22
	NO*	−1.87	0.32	<0.01	−2.69	−1.04
NO	CAM*	1.65	0.57	0.03	0.16	3.14
	NAV*	1.87	0.32	<0.01	1.04	2.69
Backward flow volume (PV) [ml], *N* = 22						
CAM	NAV	−0.10	0.08	0.64	−0.29	0.10
	NO	−0.09	0.09	0.96	−0.32	0.14
NAV	CAM	0.10	0.08	0.64	−0.10	0.29
	NO	0.01	0.10	1.00	−0.26	0.28
NO	CAM	0.09	0.09	0.96	−0.14	0.32
	NAV	−0.01	0.10	1.00	−0.28	0.26
Regurgitation fraction (PV) [ ], *N* = 22						
CAM	NAV	−0.001	0.001	1.00	−0.004	0.002
	NO	0.000	0.001	1.00	−0.003	0.004
NAV	CAM	0.001	0.001	1.00	−0.002	0.004
	NO	0.001	0.002	1.00	−0.003	0.006
NO	CAM	0.000	0.001	1.00	−0.004	0.003
	NAV	−0.001	0.002	1.00	−0.006	0.003
Velocity rate (PV) [cm/s], *N* = 660						
CAM	NAV	0.04	0.14	1.00	−0.29	0.37
	NO*	−0.54	0.16	<0.01	−0.94	−0.15
NAV	CAM	−0.04	0.14	1.00	−0.37	0.29
	NO*	−0.58	0.16	<0.01	−0.97	−0.19
NO	CAM*	0.54	0.16	<0.01	0.15	0.94
	NAV*	0.58	0.16	<0.01	0.19	0.97

* The mean difference is significant at the 0.05 level.

^a^ Adjustment for multiple comparisons: Bonferroni.

For the MV, no significant difference was found in forward flow volume (Wilks' lambda = 0.89, *F*(2,21) = 1.32, *P* = 0.29, *η* ^2^ = 0.11), backward flow volume (Wilks' lambda = 0.99, *F*(2,21) = 0.19, *P* = 0.83, *η* ^2^ = 0.02), regurgitation fraction (Wilks' lambda = 0.99, *F*(2,21) = 0.15, *P* = 0.86, *η* ^2^ = 0.01), and velocity rate (Wilks' lambda = 1.00, *F*(2,687) = 1.28, *P* = 0.28, *η* ^2^ = 0.04). The MV pairwise comparisons are listed in Table S3 and illustrated in Fig. S6 in the Supplemental Material.

For the AV, no significant difference was observed in forward flow volume (Wilks' lambda = 0.93, *F*(2,21) = 0.74, *P* = 0.49, *η* ^2^ = 0.07), backward flow volume (Wilks' lambda = 0.99, *F*(2,21) = 0.08, *P* = 0.92, *η* ^2^ = 0.01), regurgitation fraction (Wilks' lambda = 0.92, *F*(2,21) = 0.97, *P* = 0.40, *η* ^2^ = 0.08), and velocity rate (Wilks' lambda = 1.00, *F*(2,687) = 1.18, *P* = 0.31, *η* ^2^ = 0.03). The AV pairwise comparisons are listed in Table S4 and illustrated in Fig. S7 in the Supplemental Material.

For the group of insufficient valves *n*_2_, no significant difference was observed for backward flow volume (Wilks' lambda = 0.89, *F*(2,17) = 0.97, *P* = 0.40, *η* ^2^ = 0.10) and regurgitation fraction (Wilks' lambda = 0.94, *F*(2,17) = 0.55, *P* = 0.59, *η* ^2^ = 0.61). The n_2_ pairwise comparisons are listed in Table S5 and illustrated in Fig. S3 in the Supplemental Material.

**FIGURE 5:**
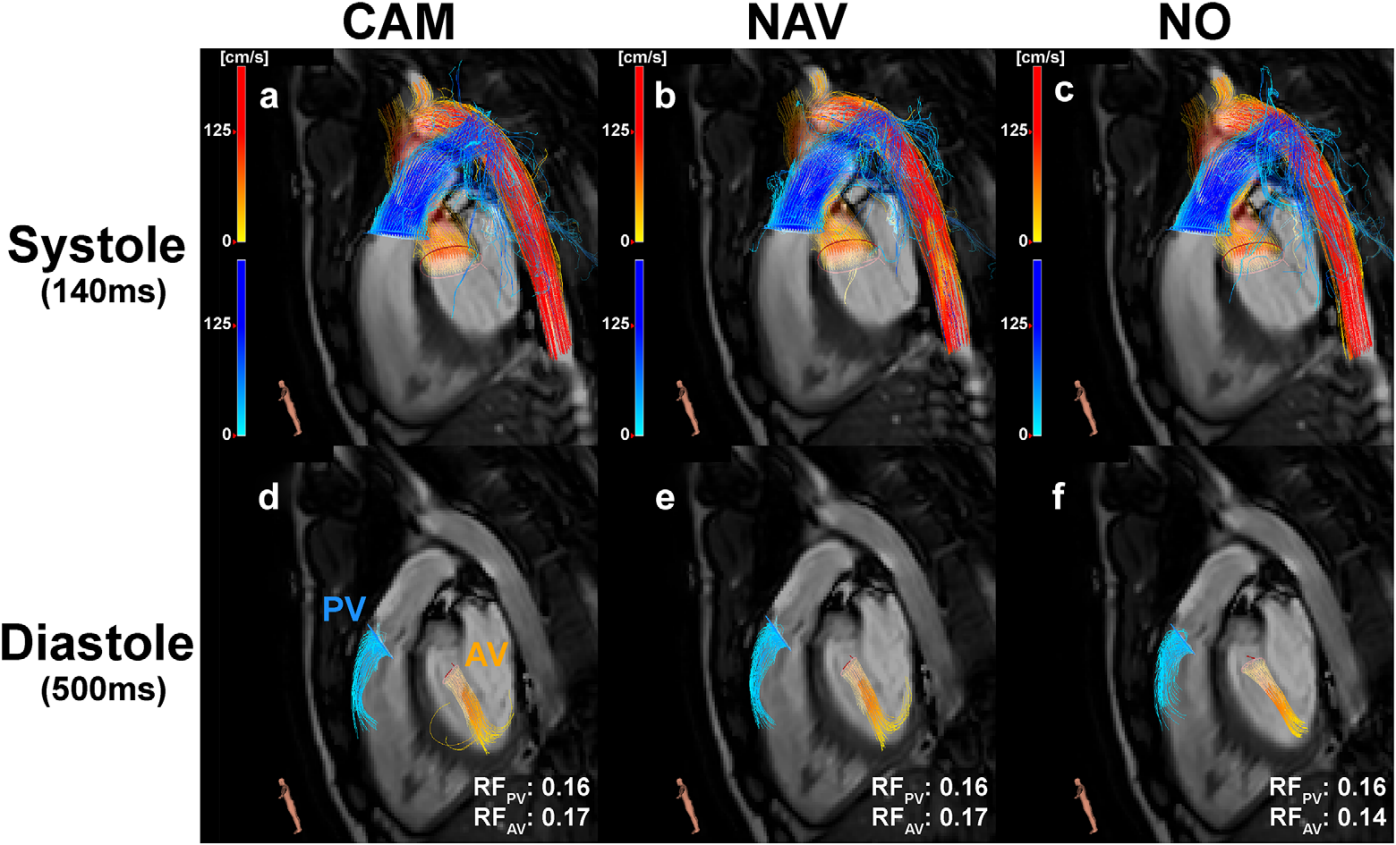
Whole heart 4D flow analysis in CAAS. Shown are streamlines of CAM, NAV, and NO data sets for both systole (a, b, c) and diastole (d, e, f). Regurgitation through the pulmonary valve (PV) and aortic valve (AV) can be seen during diastole. The corresponding regurgitation fractions (RF) are reported in (d), (e), and (f).

An example 4D flow analysis can be seen in Fig. 5 and Video S1 in the Supplemental Material showing the streamlines and regurgitation fraction for all three methods in a patient with mild PV and AV regurgitation.

## Discussion

This study has compared whole‐heart 4D flow MRI in patients with a spectrum of cardiovascular diseases using retrospective camera‐based gating, navigator‐based gating, and no gating. We observed that CAM and NAV data sets had similar image quality and flow measurements. Compared to NO, both CAM and NAV showed improvements in quantitative image quality scores. However, no difference in qualitative image quality scoring was found between NAV, CAM, and NO. In a quantitative flow analysis, significant differences were measured in transvalvular blood flow of two out of four valves for CAM‐vs‐NO and NAV‐vs‐NO.

The respiratory signals from CAM and NAV could not be directly compared by their amplitudes because of the different signal derivation of absolute liver displacement in millimeters (NAV) and an arbitrary signal (CAM). Those signal differences were noticeable in the different signal ranges or outlier peaks. Also, the signal amplitude range for CAM has a lower variation between patients compared to NAV, which did not allow for amplitude binning after prior rescaling of the signal. Therefore, although amplitude binning is superior to phase binning in terms of motion correction (for NAV),^29^ only phase binning could be used in this study to enable a fair comparison with camera‐based gating. In applications that require the information of absolute displacement in millimeter, as for instance in radiotherapy, the amplitude binned NAV is superior to CAM with an arbitrary motion signal. However, in applications in which a relative displacement is an option, such as 4D flow MRI, the derived respiratory phase is sufficient to compensate for respiratory motion, especially if the underlying signal amplitude is arbitrary as the respiratory belt, self‐gating, or camera signal. Therefore, both methods perform equally well after phase binning which can be seen in the strong correlation. The applied phase binning is robust for signal outliers and respiratory drifts (change of signal amplitude over time), which is highlighted in Fig. 2. Furthermore, the reported phase delay was zero, which indicates no phase shift or different respiratory motion estimates, respectively, between the two methods. Moreover, the reported phase delay SD is acceptable. Even for the highest respiratory rates of 40 breaths per minute (breathing cycle duration of 1500 ms), which are only applicable for newborns and elderlies,^30^, ^31^ a difference of 63 ms would result in a mismatch of 4.2% and even decrease for lower respiratory rates (2.1% for 20 breaths per minute).

The qualitative image quality assessment did not result in any significant difference between the gating methods in all categories. However, the extremely low inter‐rater reliability showed that the analysis itself had no significance and does not allow for a solid conclusion. Similarly, this result may indicate that the quality definitions were insufficient or that the raters were given poor instructions.

4D flow images are generally not high in contrast or rich in anatomical detail,^1^ which makes it challenging to identify subtle differences in a 4‐point Likert‐scale analysis. The quantitative image quality analysis with an objective measuring method showed that NAV and CAM data sets were superior to NO data sets in terms of SNR and LLE. Especially the decreased LLE (reduced blurring) for gated reconstruction underlined the benefit of respiratory gating.

The quantitative flow analysis supported the respiratory phase and image quality findings of a good overall agreement between CAM and NAV. Significant local differences were observed for NO in the valves of the right heart (TV and PV) showing that non‐gated reconstructions likely lead to impaired flow measurements. However, this cannot be generalized as significant differences were only in TV backward flow volume, TV regurgitation fraction, PV forward flow volume, as well as TV and PV, mean velocity rate; and no significant differences were observed in the other categories including the valves of the left heart (AV and MV). Moreover, the analysis of the valve insufficiency subgroup n_2_, showing no significant difference for backward flow volume and regurgitation fraction, indicates that diagnosis and risk assessment based on CAM, NAV, and NO image reconstructions will not differ. Altogether, the conclusion can be drawn that NO has noticeable flow measurement differences in valves of the right heart, and that CAM and NAV data sets show no differences overall. Three major questions might be raised when interpreting the results.

First, is respiratory gating needed, or is the expected motion perturbation without gating acceptable? In this study, a 60% expiration phase acceptance together with a spatial resolution of 2.5 mm isotropic was used and regional differences in transvalvular blood flow were observed for respiratory‐gated data sets (CAM and NAV) compared to NO data sets. Although CAM and NAV data sets had fewer data points for image reconstruction, the respiratory gating resulted in superiority compared to NO data sets. Other studies^14^, ^15^ have shown that 100% respiratory phase acceptance together with a 3.0 mm isotropic resolution, which is the largest voxel size recommended for whole‐heart 4D flow MRI,^1^ resulted in acceptable flow errors and preserved quantitative flow results. However, Dyverfeldt and Ebbers have shown that spatial resolutions finer than the degree of accepted respiratory motion do not result in improved data quality.^7^ Moreover, in the presented data the respiratory gating resulted in reduced LLE (less blurring) of about 2 voxels or 5 mm, which might be at the edge of a noticeable impact of respiratory gating as some categories have shown an effect and others did not. Therefore, these findings can be interpreted that the impact of respiratory motion is depending on the anatomy under investigation and the used voxel size. When interested in accurate flow measurements for smaller voxel sizes (<2.5 mm), the impact of respiratory motion will likely be stronger than for more coarse resolutions.

Second, is the effect of respiratory gating of clinical relevance? Although significant differences were observed for the TV and PV in the quantitative flow analysis, the differences must be put into context. For instance, the mean difference of TV backward flow volume measured with CAM compared to NO was around 1.4 l. In relation to the CAM mean backward flow volume of around 15.8 ml, this would be an 8.9% difference. Cases of larger net differences can be found in the data set too; however, the relative differences are on a similar scale. So, in theory, an inaccuracy of 5%–10% is possible and can result in a different risk assessment of for instance valve regurgitation, if the regurgitant volume is below a quantitative threshold with gating and above without, or vice versa. However, in practice, the qualitative regurgitant flow measurement is just one of many indicators. Besides parameters derived from 4D flow MRI (e.g., regurgitation, shunt, peak velocity, and flow shape), also 2D flow and multi‐chamber cine images are taken into consideration. Before concluding, the physician in charge will perform a complete risk assessment with quantitative, qualitative, and semi‐qualitative indicators, which might tolerate a 5%–10% inaccuracy in determining the regurgitant flow volume. Nevertheless, any improvement of the regurgitant flow measurement should be considered if there are no other trade‐offs involved, which was the case in this study (i.e., the same scan time).

Third, if respiratory gating is preferred, what respiratory gating method should be chosen? Both methods do not require any patient interaction and, therefore, provide equal patient comfort. One clear advantage of the navigator is the respiratory motion measurement in absolute millimeter displacement unlike the arbitrary signal of the camera. However, the navigator acquisition might disturb the intended image acquisition in the form of steady‐state disruption or image sampling gaps that occur due to the navigator sampling. Therefore, CAM might be particularly useful in balanced steady‐state free precession imaging. Another important advantage of the camera is the higher sampling rate of 20 Hz compared to the 1–2 Hz of the navigator, which ensures sufficient high sampling rates even for newborns or patients with shortness of breath.^30^, ^31^ Yet another aspect might be the usability in which the CAM has an advantage as the contact‐less design facilitates a steady signal performance without any scan operator interaction like planning the navigator on the liver‐lung border. Potential error sources for the camera in a clinical setup could be that the camera tracks another repetitive motion in the visual field like arm movement or a blanket flapping because of the air condition in the bore. Moreover, the visual field could be blocked or hindered by a head coil or other device. However, this is speculative, and the presented study did not observe a significant difference between the camera and the navigator signal, or CAM and NAV, respectively. In addition, the usage of CAM can be applied to other (imaging) modalities as well, e.g., home care vital sign monitoring.^19^

Several studies have been published on contact‐free physiological monitoring,^32^, ^33^, ^34^, ^35^, ^36^, ^37^ but they did not focus on cardiac 4D flow MRI. Harder et al. compared the same camera type and setup (abdominal imaging) to existing respiratory gating methods and reported that camera‐based respiratory triggering (prospective gating) significantly improved image quality of 3D cholangiopancreatography images compared to conventional respiratory belt triggering.^21^

### Limitations

Firstly, this study was retrospective and did not include other respiratory gating techniques or flow measurement references as additional comparisons. A simultaneous signal acquisition of a respiratory belt in combination with self‐gating would have provided additional information on optimal respiratory gating. Unfortunately, both methods were not possible in this study as the k‐space sampling was not optimized for self‐gating and no respiratory belt was used. Moreover, the origin and interpretation of the different signals ranges and extrema remain unclear. Possible explanations could be body movement or abnormal breathing such as gasping, or agonal respiration; and how the vendor‐implemented algorithms deal with abnormal breathing. As the algorithms were not available, nor were patient breathing and movement video recorded, no detailed explanation can be given and should be investigated in future research.

## Conclusion

Camera‐based respiratory gating performs as well as conventional liver‐lung‐navigator‐based respiratory gating in retrospectively gated whole‐heart 4D flow MRI. Respiratory phases of both techniques were highly correlated. Quantitative image quality analysis showed that both gating techniques were equivalent and superior to images reconstructed without respiratory gating. Quantitative flow analysis revealed local flow differences in the tricuspid and pulmonary valves in images reconstructed without respiratory gating compared to those with respiratory gating, but no differences were found between images reconstructed with camera‐based and navigator‐based respiratory gating.

## Supporting information

**Fig S1** Visualization of the CAM and NAV signal amplitudes of all patients in the form of boxplots (A) and (C) and all signals put behind each other (B) and (D) after zero mean shifting. A vertical dotted line indicates a new patient. The boxplots show the signal minima (min) and maxima (max), the data quartiles Q_1_, Q_2_ (median), Q_3_, and the upper and lower whiskers W_up_ (Q_3_ + 1.5 x (Q_3_‐Q_1_)), W_low_ (Q1–1.5 x (Q_3_‐Q_1_)).

**Fig S2** Bland–Altman plots of quantitative image analysis. The overlapping ordinal data points have been jittered for better visualization.

**Fig S3** Bland–Altman plots of qualitative image analysis (left) and quantitative flow analysis of insufficiency group n_2_.

**Fig S4** Bland–Altman plots of quantitative flow analysis of tricuspid valve.

**Fig S5** Bland–Altman plots of quantitative flow analysis of pulmonary valve.

**Fig S6** Bland–Altman plots of quantitative flow analysis of mitral valve.

**Fig S7** Bland–Altman plots of quantitative flow analysis of aortic valve

**Table S1** Patient cohort overview of diagnosis and demographics (sex, age, and weight).
**Table S2:** Pairwise comparisons of qualitative image analysis in form of anatomical structure, breathing artifacts, flow artifacts, and flow signal.
**Table S3:** Pairwise comparisons of mitral valve (MV) quantitative flow analysis in form of forward flow volume, backward flow volume, regurgitation fraction, and velocity rate.
**Table S4:** Pairwise comparisons of aortic valve (AV) quantitative flow analysis in form of forward flow volume, backward flow volume, regurgitation fraction, and velocity rate.
**Table S5:** Pairwise comparisons of valve insufficiency group n_2_ quantitative flow analysis in form of backward flow volume and regurgitation fraction.

**Video S1**. Xxx.
