# Supplementary figures and images for: Retrospective Camera‐Based Respiratory Gating in Clinical Whole‐Heart 4D Flow MRI
Source: J Magn Reson Imaging. 2021 Mar 10;54(2):440–51. doi: 10.1002/jmri.27564 (PMC8359364; doi:10.1002/jmri.27564)

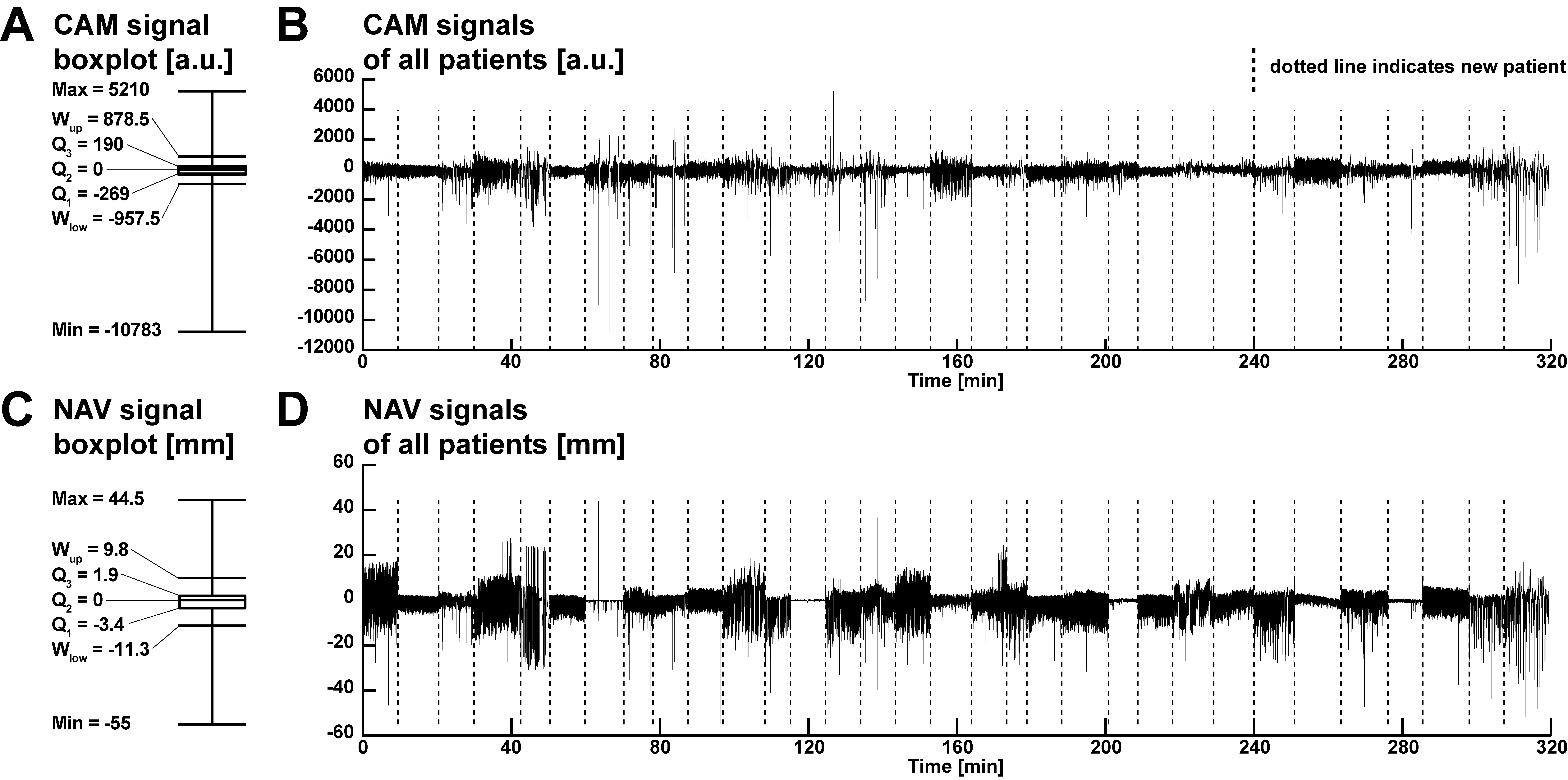

Supplement: Supplementary file 1 — Fig S1 Visualization of the CAM and NAV signal amplitudes of all patients in the form of boxplots (A) and (C) and all signals put behind each other (B) and (D) after zero mean shifting. A vertical dotted line indicates a new patient. The boxplots show the signal minima (min) and maxima (max), the data quartiles Q1, Q2 (median), Q3, and the upper and lower whiskers Wup (Q3 + 1.5 x (Q3‐Q1)), Wlow (Q1–1.5 x (Q3‐Q1)). [file JMRI-54-440-s008.tif]

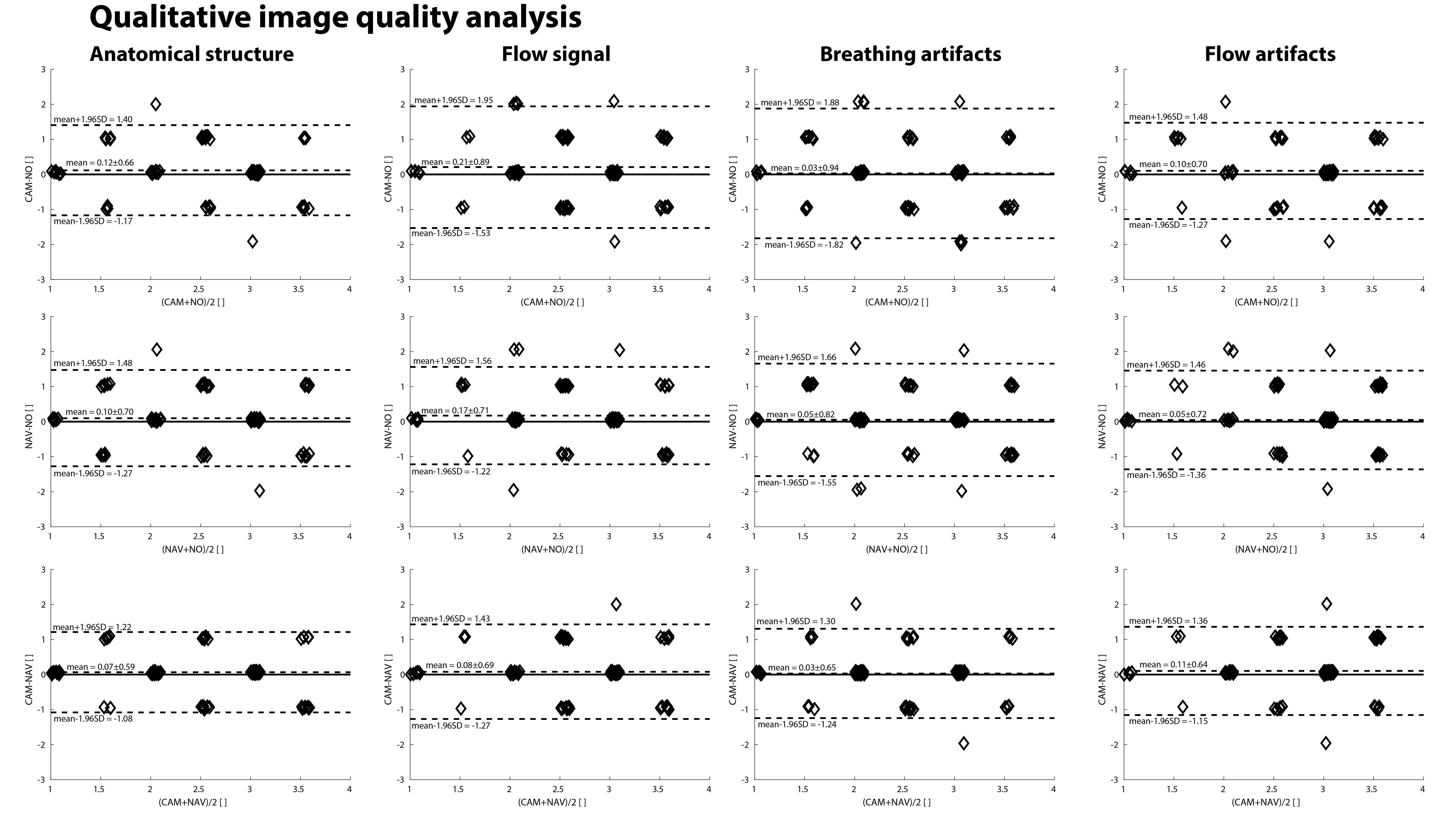

Supplement: Supplementary file 2 — Fig S2 Bland–Altman plots of quantitative image analysis. The overlapping ordinal data points have been jittered for better visualization. [file JMRI-54-440-s005.tif]

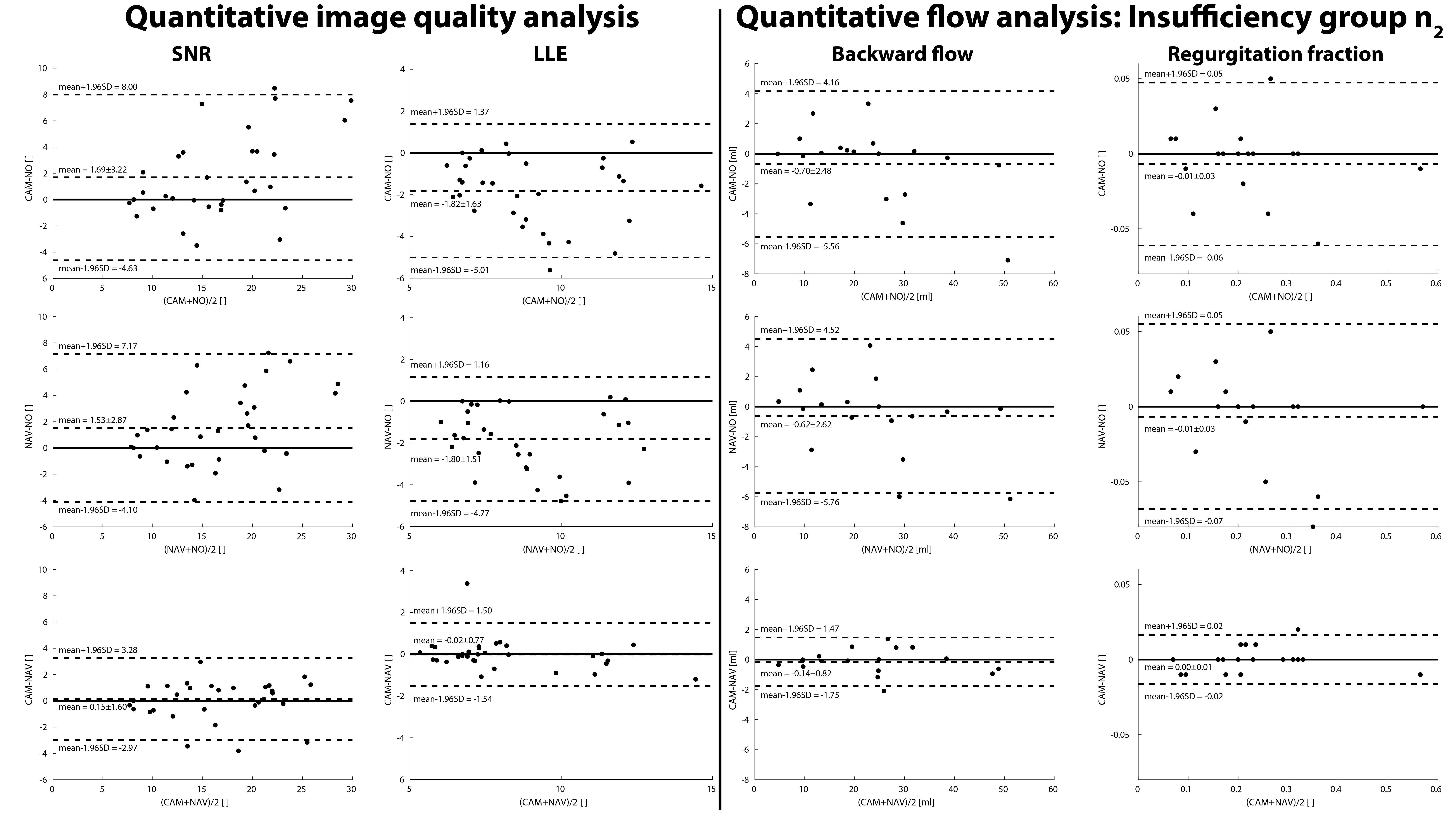

Supplement: Supplementary file 3 — Fig S3 Bland–Altman plots of qualitative image analysis (left) and quantitative flow analysis of insufficiency group n2. [file JMRI-54-440-s002.tif]

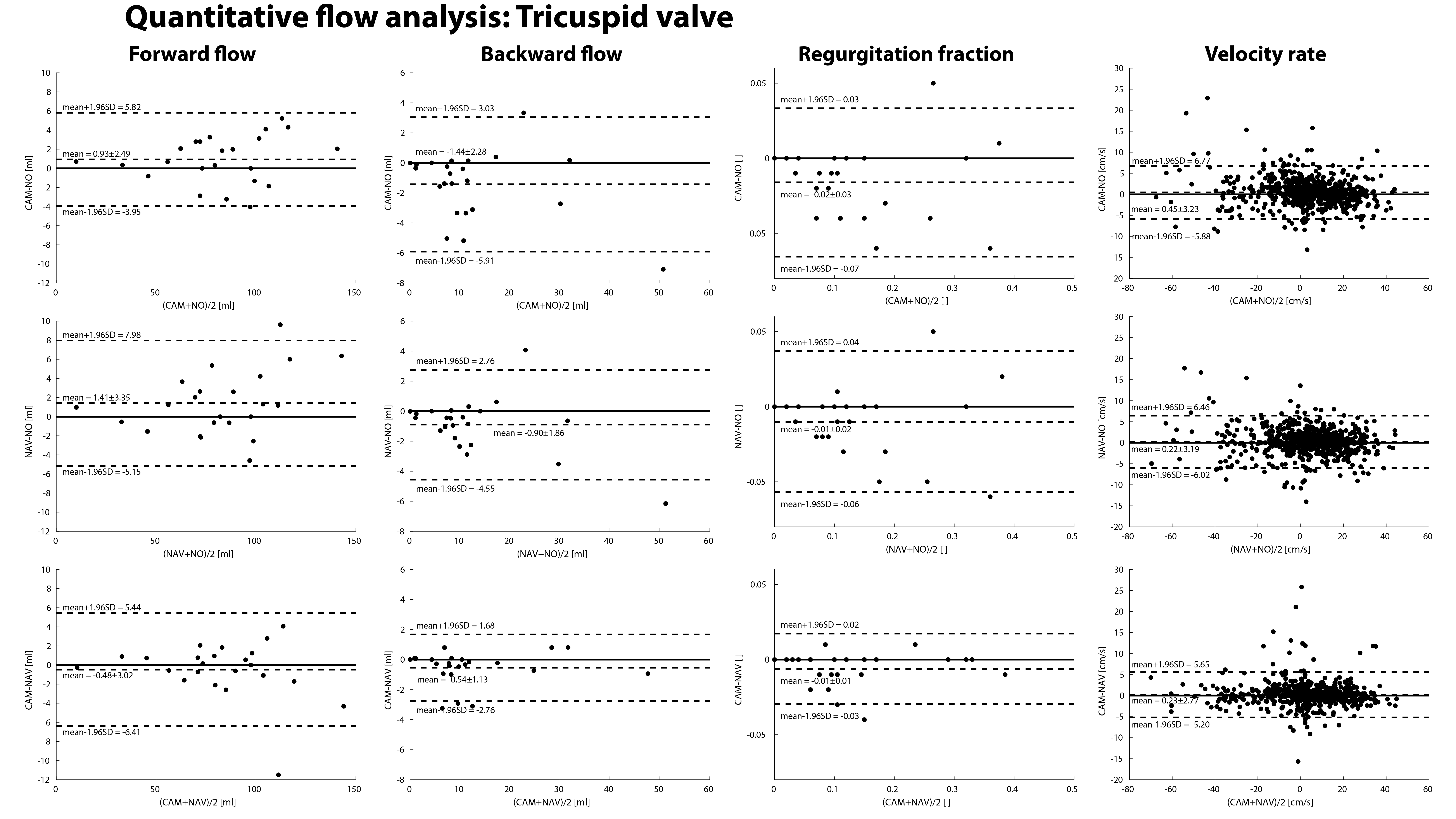

Supplement: Supplementary file 4 — Fig S4 Bland–Altman plots of quantitative flow analysis of tricuspid valve. [file JMRI-54-440-s009.tif]

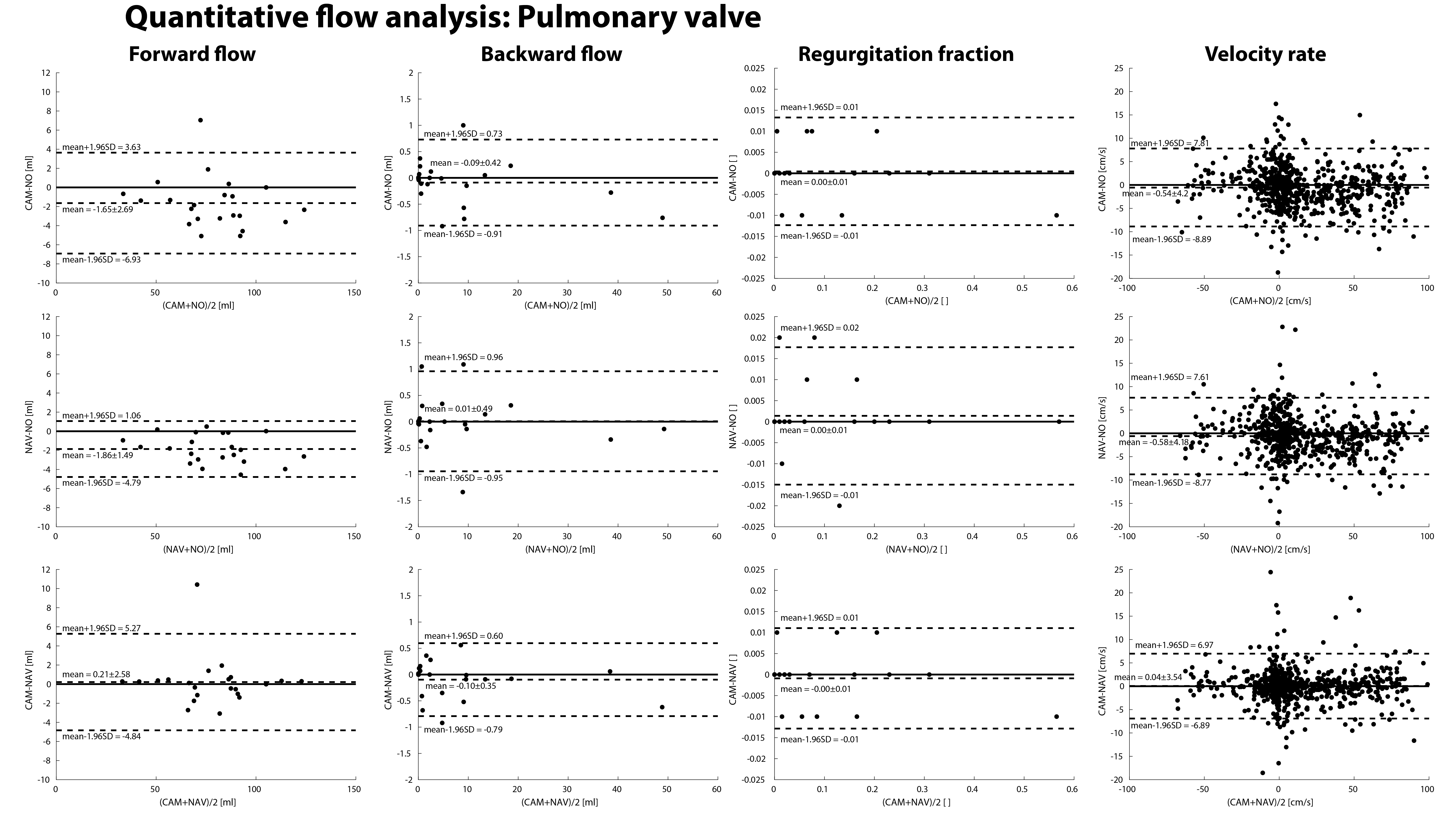

Supplement: Supplementary file 5 — Fig S5 Bland–Altman plots of quantitative flow analysis of pulmonary valve. [file JMRI-54-440-s006.tif]

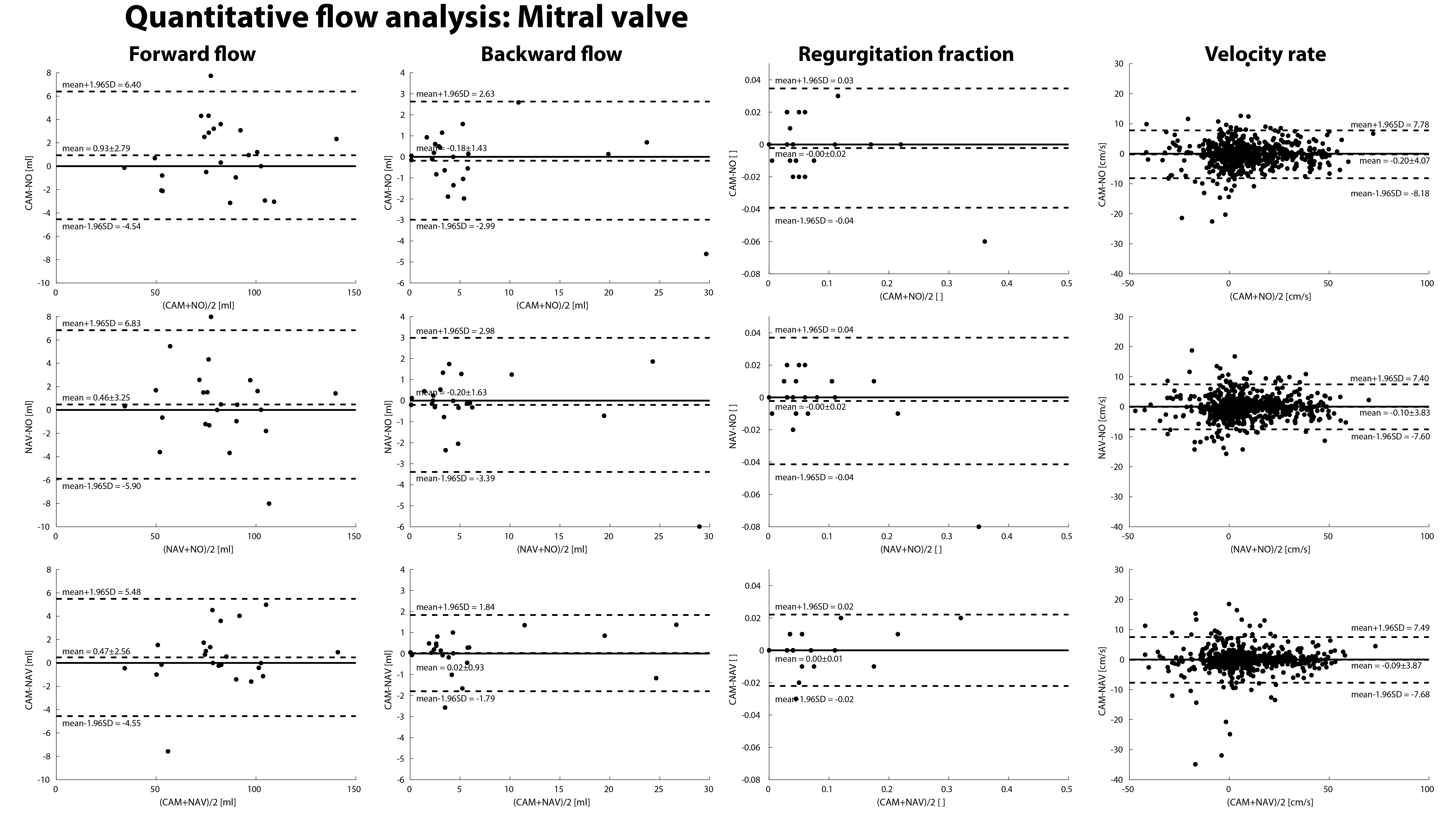

Supplement: Supplementary file 6 — Fig S6 Bland–Altman plots of quantitative flow analysis of mitral valve. [file JMRI-54-440-s004.tif]

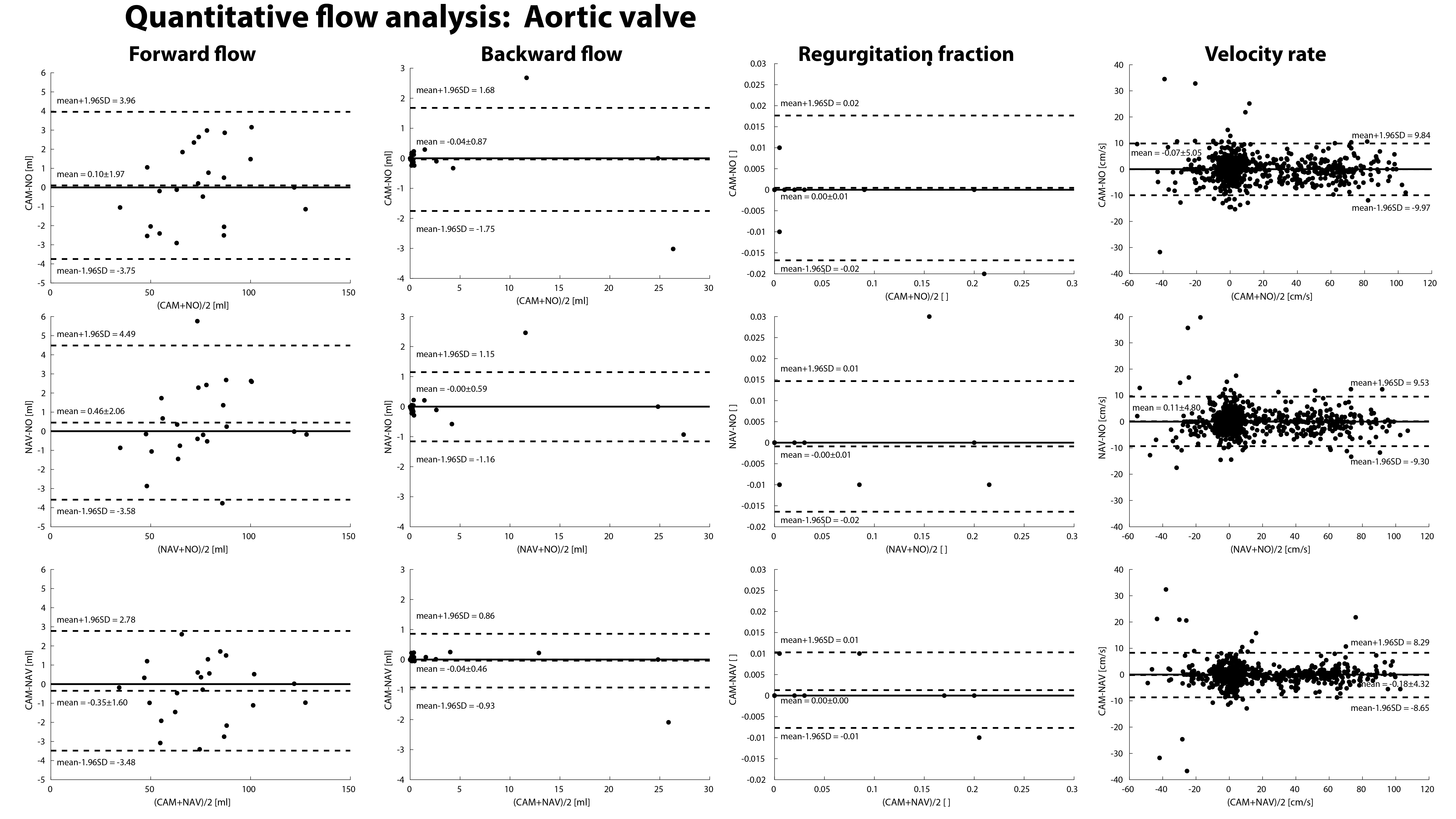

Supplement: Supplementary file 7 — Fig S7 Bland–Altman plots of quantitative flow analysis of aortic valve [file JMRI-54-440-s003.tif]
